# Computational Modeling of the Size Effects on the Optical Vibrational Modes of H-Terminated Ge Nanostructures

**DOI:** 10.3390/molecules18044776

**Published:** 2013-04-22

**Authors:** Alejandro Trejo, Miguel Cruz-Irisson

**Affiliations:** Instituto Politécnico Nacional, ESIME-Culhuacan, Av. Santa Ana 1000, DF 04430, Mexico; E-Mail: atrejob0800@ipn.mx

**Keywords:** porous germanium, germanium nanowires, phonon confinement, DFPT

## Abstract

The vibrational dispersion relations of porous germanium (pGe) and germanium nanowires (GeNWs) were calculated using the *ab initio* density functional perturbation theory with a generalized gradient approximation with norm-conserving pseudopotentials. Both pores and nanowires were modeled using the supercell technique. All of the surface dangling bonds were saturated with hydrogen atoms. To address the difference in the confinement between the pores and the nanowires, we calculated the vibrational density of states of the two materials. The results indicate that there is a slight shift in the highest optical mode of the Ge-Ge vibration interval in all of the nanostructures due to the phonon confinement effects. The GeNWs exhibit a reduced phonon confinement compared with the porous Ge due to the mixed Ge-dihydride vibrational modes around the maximum bulk Ge optical mode of approximately 300 cm^−1^; however, the general effects of such confinements could still be noticed, such as the shift to lower frequencies of the highest optical mode belonging to the Ge vibrations.

## 1. Introduction

Recently, germanium (Ge) nanomaterials, such as porous Ge (pGe) and Ge nanowires (GeNWs), have become attractive due to the higher electron mobility of Ge with respect to silicon. Multiple potential applications have thus arisen for such nanostructures in the microelectronics field, such as in anodes in lithium ion batteries [1,2], backside reflectors in thin film solar cells [3], new fabric materials [4], and field effect transistors [5]. The quantum confinement and surface effects in nanopores and nanowires is of great importance [6,7] because these effects modify the electronic and chemical properties of the nanomaterials with increased band gaps and the presence of photoluminescence in nanostructures developed from indirect gap semiconductors, such as Si or Ge. There has been important advances in the synthesis of nanostructures in the few nm range where ultrathin Si, ZnO and Ge nanowires have been reported [8,9,10,11]. An important feature of nanopores and nanowires is the special relationship between them as the pore diameter increases to the percolation limit (*i.e.*, when the pores touch each other); the product is a network of nanowires such as the one synthesized by Fang and coworkers [12]. In addition, the porous case has been treated as a collection of interconnected nanowires, and the effects of such interconnection on the electronic and optical properties of nanopores and nanowires has been examined in other theoretical works [13,14]. Another important quantum mechanical feature of nanopores and nanowires is the phonon confinement effect because many material properties, such as thermal transport, can be understood in terms of phonons. A phonon is defined as quanta of crystal lattice vibrations; these vibrations affect the physical processes in solids. The phonon confinement can be understood as follows: phonon propagate in a crystal lattice with energy dependent to the wavevector (***q***) in the Brillouin zone (BZ), since the BZ is much larger than the scattered light in optical spectroscopic techniques and as consequence of momentum conservation, only the phonons on the BZ center (***q*** = 0) can contribute to their spectrum specially the Raman response. When a system is confined such as in nanowires, the phonon wave function decays near the boundary of the nanostructure, this restriction on the spatial extent of the wavefunction, via a relationship of the uncertainty principle type, leads to discrete values of wave vector ***q***, of which the smallest ***q*** is π/*d*, and its multiples, where *d* is the size of the crystal. The selection rules are relaxed by these effect and some modes other than the ones at the BZ center are taken into account in the spectrum, which is reflected through an asymmetric broadening and a red-Shift of the highest peaks in the vibrational spectrum [15,16]. In the nanopore case, something similar happens, however there is only a partial confinement which is due to extra nodes in the phonon wave function at the boundary of the pores, allowing only wavelengths smaller than the separation between pores, which produces a shift of the highest optical modes towards lower frequencies [17]. Phonon confinement effects have been observed through optical spectroscopic techniques, such as Raman scattering [7]. However, there have been only a few theoretical works that attempted to characterize the vibrational properties of these materials [18,19]. In this paper, we studied the phonon density of states of Ge nanostructures with the first principles density functional perturbation theory technique using the generalized gradient approximation and norm-conserving pseudopotentials. We performed a comparison of the phonon confinement effects between porous structures and nanowires by comparing their respective density of states with similar confinement distances.

## 2. Model and Calculation Scheme

**Figure 1:**
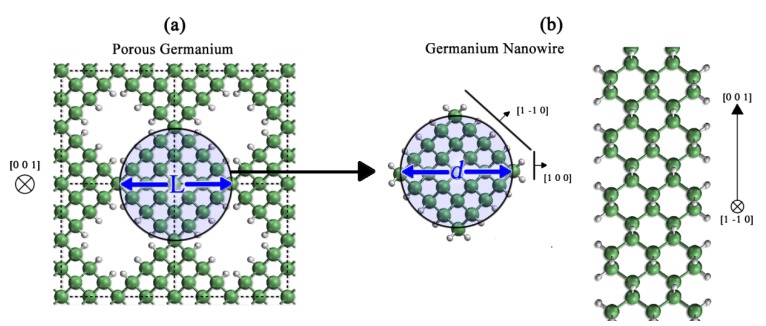
Models of Ge nanostructures. (**a**) Top view of an 8-atom supercell of pGe with p = 12.5%, where the circle shows the confined area; (**b**) top and side views of a GeNW grown in the [001] direction. The green and grey spheres represent Ge and H atoms, respectively.

The nanostructures were modeled using the supercell technique [20,21]. In the case of the pGe, columns of Ge atoms were removed from an otherwise perfect Ge crystal along the [001] direction, taking special care that the pore conserved a rhombus shape. To study the effects of morphology and porosity on the vibrational properties of the pGe, the pores were modeled in two supercells: the first was an eight-atom cell with the lattice parameter of crystalline Ge (*a* = 5.65 Å), and the second was a thirty-two-atom supercell built by the union of four eight-atom cells. The porosity (p) in this case is defined as the ratio between the number of atoms removed and the total number of atoms in the original supercell, and the confinement distance is the diameter of a circle that encloses the Ge portion of the material and has the pore in its axis (L) (Figure 1a). For the nanowire in the [001] direction, the structures were created by removing the atoms that were outside of a circumference defined by a diameter *d*. In this case, the periodicity of the system is that of the crystalline Ge lattice parameter (*c* = 5.65 Å). The [001] direction was chosen because the pGe could be regarded as a collection of interconnected nanowires [13] at the percolation limit; the effect of said interconnection on the vibrational properties of these structures has yet to be studied.

The density of states (DOS) of the pGe and the GeNWs was calculated by means of the *ab initio* density functional perturbation theory (DFPT) [22] with the generalized gradient approximation (GGA) using the CASTEP code [23] implemented in the Materials Studio Software Suite. The GGA function that was used was the Perdew-Burke-Ernzerhof (PBE) [24] using norm-conserving pseudopotentials. The Brillouin zone of each nanostructure was sampled to a highly converged set of k-points with grids up to 1 × 1 × 6 according to the Monkhorst-Pack scheme [25]. The surface dangling bonds of all of the nanostructures were saturated with H atoms to remove states within the electronic energy band gap because there are experimental indications of hydride termination in the surface of these nanostructures [26,27], although there is experimental evidence of H in the surface of the nanostructures, it is worth noticing that this is a highly idealized model, since surface defects and other chemical species attached to the pores and wires surfaces are expected, which would modify drastically the phonon spectrum of the nanostructures, however we are using these model as a first approach to the surface of the nanostructure which is interesting for comparative purposes for future works which involve different surface defects, that are out of reach to the current work. All of the nanostructures were relaxed with the BFGS algorithm [28] to obtain their minimum energy configurations and to avoid imaginary frequencies in the vibrational spectrum, which are the product of a badly converged optimization (*i.e.*, the transitional state minimum instead of the absolute minimum energy state). Our convergence parameters were such that the maximum forces were 0.01 eV/Å, the maximum cell stress was 0.02 GPa, and the convergence for the electronic energy was achieved with a tolerance of 5E-7 eV.

## 3. Results and Discussion

**Figure 2:**
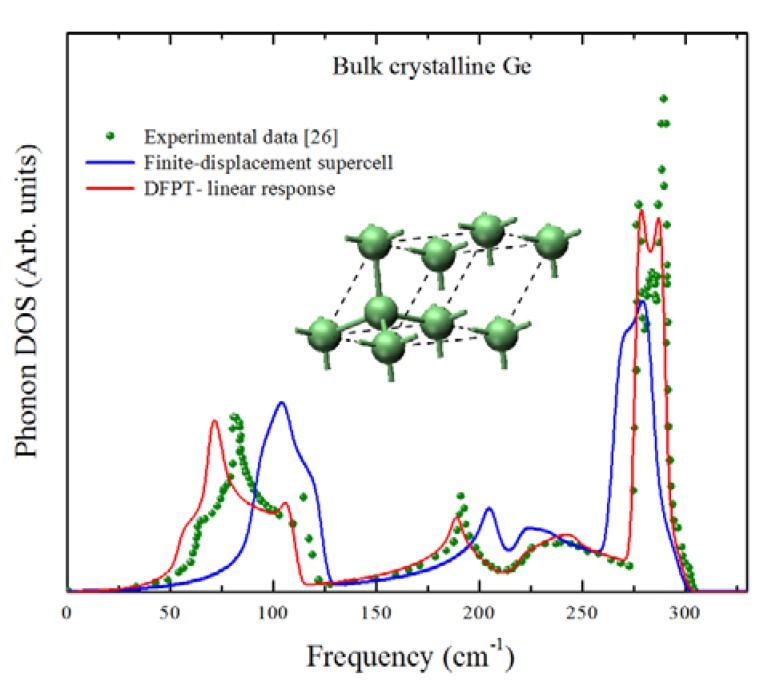
The phonon density of states (DOS) of crystalline Ge using two first principles approaches, finite displacement-supercell (blue line) and DFPT (red line), compared with the experimental results taken from [33].

In Figure 2, we show the phonon density of states (DOS) of crystalline Ge calculated by using two algorithms: the finite displacement supercell scheme [29,30] and the density functional perturbation theory linear response approach [31,32], both calculation results are compared with the experimental data from reference [33]. The finite displacement results were obtained considering a force constant cutoff of 10 Å which, after a series of tests with increasing cutoffs, was chosen so the highest optical interval of the spectrum had the best fit possible regarding the peaks positions. It can be observed that, although both approaches yield results that are in reasonably good agreement with the experimental data especially near the highest optical mode (approximately 300 cm^−1^), the DFPT results have a better fit with the experimental results in the remaining portion of the spectrum. We thus decided to use the linear response approach for our calculations.

**Figure 3:**
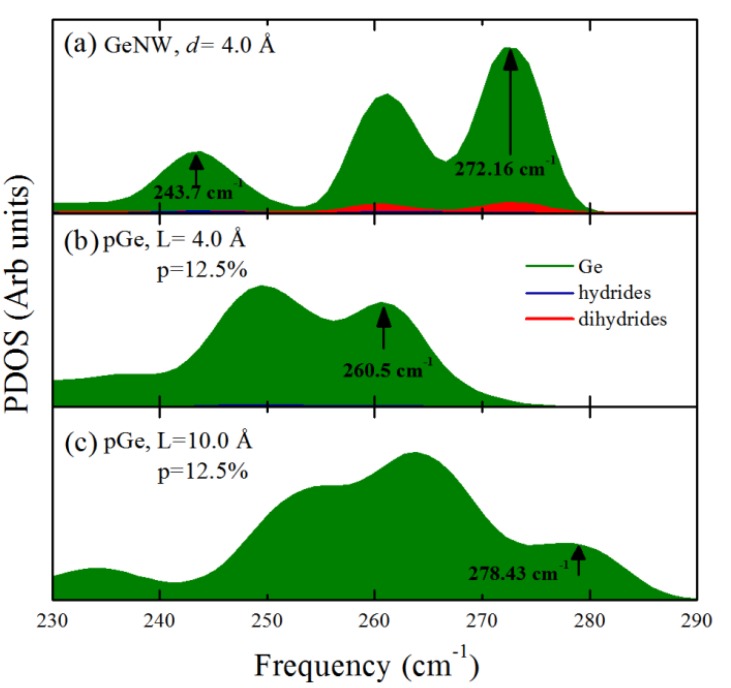
Partial phonon density of states (PDOS) of (**a**) GeNW with *d* = 4.0 Å, (**b**) pGe with L = 4.0 Å and p = 12.5%, and (**c**) pGe with L = 10.0 Å and p = 12.5%. The green, red and blue areas represent Ge, hydride and dihydride vibrations, respectively.

Once the phonon calculation scheme was selected, we calculated the partial phonon density of states (PDOS) of the nanowires and nanopores. In Figure 3, we show that the PDOS of (a) a GeNW with a diameter of 4.0 Å compared with (b) pGe with p = 12.5% and the same approximate confinement distance of 4.0 Å (*L≈d*). It can be observed that the expected behavior is not observed; a greater shift of the highest optical modes toward lower frequencies in the case of the nanowire compared with the porous case was expected since the nanowire has a total confinement along its cross section, while the porous case has only a partial confinement where the phonon wave functions are extended states but have extra nodes due to the pore presence, located at the pore boundary, these nodes determine the highest modulation of phonon wavelength, allowing only wavelengths smaller than *L*. Instead, we observed that there are higher optical modes in the nanowire compared with the porous case. It was postulated that the extra modes could be caused by the bending vibrations of the Ge-dihydride (H-Ge-H where the red area in Figure 3 represents the contribution of dihydride bonded hydrogen to the DOS) groups at the surface of the nanowires, such modes could mask the quantum confinement features in the nanowire and produce a greater shift in the porous case because this pore morphology avoids the presence of Ge-dihydride bonds in its surface. The frequency of these particular Ge-dihydride bending modes could be the result of a hybridization due to the proximity of such bonds since the corners of the nanowires are too close together. However, when compared with the highest frequency modes that exhibit no dihydride contribution, the shift in the nanowire (243.7 cm^−1^) is noticeably larger than the shift in the nanopore (260.5 cm^−1^). Along with the phonon confinement one important feature that modifies the behavior of the phonon DOS is the capillary stress due to the curved surfaces of the nanostructures, the large surface to core ratio increases the number of surface states compared to the core states as the nanowire diameter decreases, which reduces the optical frequency mode weight, these effects are accounted in [34]. To corroborate the nature of the observed effects, the 4.0 Å nanopore was compared with a nanopore with the same relative porosity but with a lower confinement distance (L = 10 Å). It can be observed that when the Ge-dihydride contributions are not present, the phonon confinement effects are more apparent because the highest optical modes in the 10 Å pore are less shifted (278.43 cm^−1^) compared with the 4.0 Å case (260.5 cm^−1^), which could also be explained in the surface states scheme as the surface to core ratio decreased thus decreasing the number of surface states. This result suggests that although the phonon confinement features always affect the vibrational properties of the nanostructures, the surface also contributes to a large extent to the vibrational properties near the highest optical modes. Furthermore, the presence of different types of bonds, even with the same element (in this case H), can substantially modify the phonon DOS of such structures.

**Figure 4:**
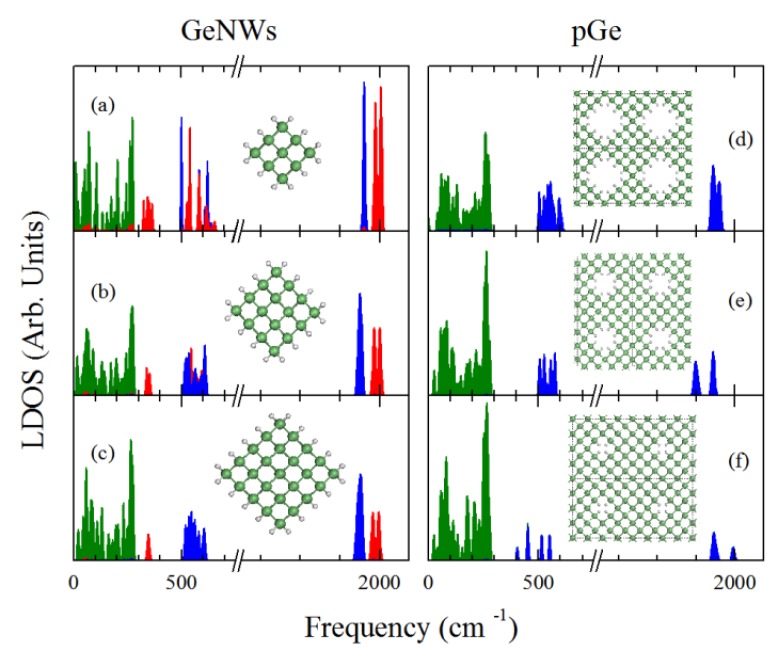
PDOS of GeNWs with a *d* of (**a**) 4 Å, (**b**) 6 Å, (**c**) 8 Å, and pGe with a p, L of (**d**) 28.125%, 8 Å, (**e**) 12.5%, 10 Å and (**f**) 3.125%, 12 Å. The green, blue and red areas represent Ge, hydride, and dihydride contributions, respectively.

To further investigate the differences between the Ge pores and nanowires with regard to their phonon spectrum, we studied the PDOS of three different nanowires compared with three diverse porosities; the results are summarized in Figure 4. To address the variation in the vibrational frequencies depending on the surface H bonds, we show a PDOS denoted by three areas: the green area represents the contribution of the Ge atoms, while the blue and red areas represents the contribution of single hydride H, and dihydride H (H atoms of H-Ge-H bonds) to the DOS respectively. In the nanowires case it can be observed that in the energy range between 300 and 500 cm^−1^, there is a red area that decreases its width while the nanowire diameter increases, which correspond to bending vibrational modes in the Ge-dihydrides. The gradual narrowing of this area is due to the lower number of dihydride bonds relative to single hydride bonds, also due to the lower hybridization between dihydride phonon modes as the nanowire corners are separated when the diameter increases. This feature allows for a clearer phonon confinement signature on the larger diameter nanowires compared with the smaller ones since the shift of the highest optical modes to lower frequencies is not masked by the dihydride vibrations. A second region can be observed in the nanowire case (at approximately 500 to 750 cm^−1^), which is comprised of dihydride and hydride vibrations and no contribution from the Ge; this region consists of bending modes. As the hydride/dihydride ratio increases, the hydrogen bending region becomes mostly dominated by single hydride vibrations, which can be expected due to the large number of single hydrides. Finally, a fourth region can be identified (around 2000 cm^−1^), which is composed of stretching vibrations of single hydrides and dihydrides. It can be observed that even when the nanowire diameter increases the single hydride and dihydride contributions are clearly distinguished, which could be important to the characterization of such nanowires with the different spectroscopy techniques. As for the nanopore case, only three regions could be identified (expected due to the absence of the dihydrides at the surface): the Ge dominated vibration region (0 to 300 cm^−1^), the single hydride bending interval (400 to 750 cm^−1^), and the H stretching (2000 cm^−1^) modes. It is important to note that the results shown in Figure 4c,d have the same relationship as those depicted in Figure 3a,b but with *L≈d* of 8 Å. When comparing the Ge contribution (0–300 cm^−1^) in the cases of Figure 4c,d the effects of the dihydrides are reduced due to the increased concentrations of Ge-hydride bonds, hence greater shift of the highest optical modes in the case of the nanowire was observed, according to quantum confinement scheme. Other possible explanation of the decreased contribution of the dihydrides to the DOS around 300 cm^−1^ in the 8 Å NW case, could be due to the separation between the dihydrides. In the lower diameter NWs the corners are too close hence a phonon hybridization could take place, thus shifting to lower energies the dihydride rocking modes (normally at 500 cm^−1^ for Ge dihydride bonds [35]); as the nanowire diameter increases these modes become less hybridized shifting to their normal interval of frequencies (400 to 500 cm^−1^). Regardless the dihydride behavior it can be observed that when comparing this set of nanowires with nanopores in a 32 atom supercell; the effect of the phonon confinement is observed since the last highest optical modes with Ge contribution of the GeNW were shifted to a lower frequency compared with that of the pGe.

**Figure 5:**
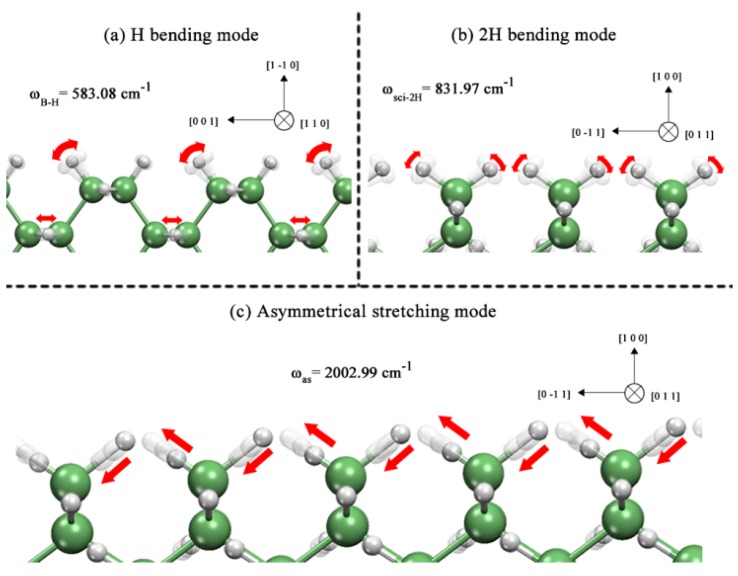
GeNWs vibration modes of (**a**) single hydride bending mode (*ω_B-H_*), (b) dihydride scissor-bending mode (*ω_sci-2H_*) and (c) dihydride asymmetrical stretching mode (*ω_as_*).

Finally, we diagrammed the surface vibrations of the atoms in the nanowire case because the porous surface is composed only of single hydrides that are also present in the nanowire case and show a similar displacement. First, the single hydride bending vibration for the 6 Å nanowire is shown in Figure 5a. It can be observed that, in this particular mode, a single hydride is performing a bending movement that is normal to the bond axis; this movement does not perturb the adjacent dihydride to a great extent, hence it can be regarded as a pure H bending vibration, which can be identified through spectroscopy techniques, such as infrared. Similar observations can be made for the following bending mode that is depicted in Figure 5b, in which a dihydride follows a scissor vibration by bending its bonds toward one direction and then to the opposite direction creating a scissor-like vibration, which is commonly studied with infrared spectroscopy in molecules such as water. Last, we present a stretching vibration along the bond axis of the dihydrides, which is called asymmetrical stretching. This stretching vibration is due to the phased motion that the atoms show, *i.e*., as one bond stretches the other bond is compressed. This asymmetrical stretching mode can also be identified through infrared spectroscopy.

## 4. Conclusions

In summary, we studied the vibrational properties of porous germanium and germanium nanowires with the first principles density functional perturbation theory. We calculated the phonon DOS of crystalline Ge with finite displacement and linear response DFPT methods, where the DFPT showed a better fit with experimental results. For the nanowire and nanopore case no experimental results were found for comparison since this is a first model of surface, which does not consider impurities, dislocations and other kind of defects in the surface passivation. We analyzed the difference in the phonon confinement effect between the nanowires and nanopores by comparing similar confinement distances in their cross sections (*L* ≈ *d*). However this comparison should be taken with care since in the porous case the phonon confinement is partial while in the NWs there is a total confinement. The results demonstrate that the vibrational properties are greatly influenced by the surface states of the nanostructures, which could in some instances mask the effects of the phonon confinement. This masking effect was observed in the 4 Å NW, in which surface dihydrides (H-Ge-H) introduce various vibrational modes around the maximum optical mode frequency of the crystalline Ge. When comparing nanowires and nanopores with similar diameter and confinement wall of *d* ≈ *L* ≈ 8 Å, the dihydride bonds become fewer in number relative to the single hydride bonds at the surface of nanowires, the effect of the phonon confinement becomes clearer since the highest optical modes frequencies in the nanowires shift to a lower value compared with the nanopores; this result is in concordance with the higher quantum confinement of the nanowires.

## References

[1] Song T., Jeon Y., Samal M., Han H., Park H., Ha J., Yi D.K., Choi J.-M., Chang H., Choi Y.-M., Paik U. (2012). A Ge inverse opal with porous walls as an anode for lithium ion batteries. Energy Environ. Sci..

[2] Xiao Y., Cao M., Ren L., Hu C. (2012). Hierarchically porous germanium-modified carbon materials with enhanced lithium storage performance. Nanoscale.

[3] Rojas E.G., Hensen J., Carstensen J., Föll H., Brendel R. (2011). Lift-off of Porous Germanium Layers. J. Electrochem. Soc..

[4] Holmberg V.C., Bogart T.D., Chockla A.M., Hessel C.M., Korgel B.A. (2012). Optical Properties of Silicon and Germanium Nanowire Fabric. J. Phys. Chem. C.

[5] Shin S.-K., Huang S., Fukata N., Ishibashi K. (2012). Top-gated germanium nanowire quantum dots in a few-electron regime. Appl. Phys. Lett..

[6] Tutashkonko S., Boucherif A., Nychyporuk T., Kaminski-Cachopo A., Arès R., Lemiti M., Aimez V. (2013). Mesoporous Germanium formed by bipolar electrochemical etching. Electrochim. Acta.

[7] Jalilian R., Sumanasekera G., Chandrasekharan H., Sunkara M. (2006). Phonon confinement and laser heating effects in Germanium nanowires. Phys. Rev. B.

[8] Zhang J.J., Katsaros G., Montalenti F., Scopece D., Rezaev R.O., Mickel C., Rellinghaus B., Miglio L., de Franceschi S., Rastelli A. (2012). Monolithic Growth of Ultrathin Ge Nanowires on Si(001). Phys. Rev. Lett..

[9] Stichtenoth D., Ronning C., Niermann T., Wischmeier L., Voss T., Chien C.-J., Chang P.-C., Lu J.G. (2007). Optical size effects in ultrathin ZnO nanowires. Nanotechnology.

[10] Irrera A., Artoni P., Iacona F., Pecora E.F., Franzo G., Galli M., Fazio B., Boninelli S., Priolo F. (2012). Quantum confinement and electroluminescence in ultrathin silicon nanowires fabricated by a maskless etching technique. Nanotechnology.

[11] Cademartiri L., Ozin G.A. (2009). Ultrathin Nanowires-A Materials Chemistry Perspective. Adv. Mater..

[12] Fang C., Föll H., Carstensen J. (2006). Long Germanium Nanowires Prepared by Electrochemical Etching. Nano Lett..

[13] Miranda A., Trejo A., Canadell E., Rurali R., Cruz-Irisson M. (2012). Interconnection effects on the electronic and optical properties of Ge nanostructures: A semi-empirical approach. Physica E.

[14] Rurali R., Sune J., Cartoixa X. (2007). Ordered arrays of quantum wires through hole patterning: *ab*
*initio* and empirical electronic structure calculations. Appl. Phys. Lett..

[15] Arora A.K., Rajalakshmi M., Ravindran T.R., Sivasubramanian V. (2007). Raman spectroscopy of optical phonon confinement in nanostructured materials. J. Raman Spectrosc..

[16] Osswald S., Mochalin V., Havel M., Yushin G., Gogotsi Y. (2009). Phonon confinement effects in the Raman spectrum of nanodiamond. Phys. Rev. B.

[17] Alfaro P., Cisneros R., Bizarro M., Cruz-Irisson M., Wang C. (2011). Raman scattering by confined optical phonons in Si and Ge nanostructures. Nanoscale.

[18] Peelaers H., Partoens B., Peeters F.M. (2009). Phonons in Ge nanowires. Appl. Phys. Lett..

[19] Trejo A., Cuevas J.L., Vázquez-Medina R., Cruz-Irisson M. (2012). Phonon band structure of porous Ge from *ab initio* supercell calculation. Microelectron. Eng..

[20] Cuevas J.L., Trejo A., Calvino M., Carvajal E., Cruz-Irisson M. (2012). *Ab-initio* modeling of oxygen on the surface passivation of 3CSiC nanostructures. Appl. Surf. Sci..

[21] Trejo A., Calvino M., Ramos E., Cruz-Irisson M. (2012). Computational simulation of the effects of oxygen on the electronic states of hydrogenated 3C-porous SiC. Nanoscale Res Lett.

[22] Refson K., Tulip P.R., Clark S.J. (2006). Variational density-functional perturbation theory for dielectrics and lattice dynamics. Phys. Rev. B.

[23] Clark S.J., Segall M.D., Pickard C.J., Hasnip P.J., Probert M.I.J., Refson K., Payne M.C. (2005). First principles methods using CASTEP. Z. Kristallogr..

[24] Perdew J.P., Burke K., Ernzerhof M. (1996). Generalized Gradient Approximation Made Simple. Phys. Rev. Lett..

[25] Monkhorst H.J., Pack J.D. (1976). Special points for Brillouin-zone integrations. Phys. Rev. B.

[26] Adhikari H., McIntyre P.C., Sun S., Pianetta P., Chidsey C.E.D. (2005). Photoemission studies of passivation of germanium nanowires. Appl. Phys. Lett..

[27] Buriak J.M. (2002). Organometallic Chemistry on Silicon and Germanium Surfaces. Chem. Rev..

[28] Pfrommer B.G., Côté M., Louie S.G., Cohen M.L. (1997). Relaxation of Crystals with the Quasi-Newton Method. J. Comput. Phys..

[29] Ackland G.J., Warren M.C., Clark S.J. (1997). Practical methods in *ab initio* lattice dynamics. J. Phys. Condens. Matter.

[30] Montanari B., Harrison N.M. (2002). Lattice dynamics of TiO_2_ rutile: Influence of gradient corrections in density functional calculations. Chem. Phys. Lett..

[31] Baroni S., de Gironcoli S., Dal Corso A., Giannozzi P. (2001). Phonons and related crystal properties from density-functional perturbation theory. Rev. Mod. Phys..

[32] Gonze X. (1995). Adiabatic density-functional perturbation theory. Phys. Rev. A.

[33] Nelin G., Nilsson G. (1972). Phonon Density of States in Germanium at 80 K Measured by Neutron Spectrometry. Phys. Rev. B.

[34] Meyer R., Comtesse D. (2011). Vibrational density of states of silicon nanoparticles. Phys. Rev. B.

[35] Glockling F. (1969). The Chemistry of Germanium.

